# Diabetic Heart Failure with Preserved Left Ventricular Ejection Fraction: Review of Current Pharmacotherapy

**DOI:** 10.1155/2022/3366109

**Published:** 2022-03-07

**Authors:** Jakub Benko, Matej Samoš, Tomáš Bolek, Dana Prídavková, Jakub Jurica, Martin Jozef Péč, Peter Galajda, Marián Mokáň

**Affiliations:** Department of Internal Medicine I, Jessenius Faculty of Medicine in Martin, Comenius University in Bratislava, Martin, Slovakia

## Abstract

Diabetes is associated with several diabetic-related abnormalities which increase the risk of onset or worsening of heart failure. Recent studies showed that the majority of diabetic patients present with heart failure with preserved ejection fraction (HFpEF), and the prevalence of HFpEF in diabetics is alarming. Moreover, outcomes in HFpEF are poor and could be compared to those of heart failure with reduced ejection fraction (HFrEF), with 1-year mortality ranging between 10 and 30%. In contrast to HFrEF, there is very limited evidence for pharmacologic therapy in symptomatic patients with preserved ejection fraction, and therefore, the optimal selection of treatment for diabetic HFpEF remains challenging. This narrative review article summarizes the currently available data on the pharmacological treatment of HFpEF in patients with diabetes.

## 1. Introduction

Cardiovascular diseases (CVD) and heart failure represents an important clinical problems in patients with type 2 diabetes (T2D), with reported prevalence of CVD 6.9–40.8%, reported prevalence of heart failure 4.3-21.0%, and in-hospital CVD-related mortality of 5.6–10.8% [1]. A previous screening of the prevalence of heart failure in outpatients with T2D showed a 2.4% prevalence of heart failure with reduced left ventricular ejection fraction (HFrEF) and a 17.5% prevalence of heart failure with preserved ejection fraction (HFpEF) in screened population [2]. Thus, the majority of T2D patients present with HFpEF, at least at early stages, and the prevalence of HFpEF in diabetics is alarming. In contrast to HFrEF [3, 4], there is very limited evidence for pharmacologic therapy in symptomatic patients with preserved ejection fraction, and therefore, the optimal selection of treatment for diabetic HFpEF (DHFpEF) remains challenging. This narrative review article summarizes the currently available data on the pharmacological treatment of DHFpEF.

## 2. Diabetic Heart Failure with Preserved Ejection Fraction as a Challenging Clinical Problem

Diabetes is associated with several diabetic-related abnormalities, such as ischemia from either coronary artery atherosclerosis, or microvascular dysfunction, myocardial hypertrophy, dysfunction of mitochondria, dysfunction of autonomic nervous system, proinflammation, and increased retention of sodium (upregulation of sodium-glucose cotransporters) which increase the risk of onset or worsening of heart failure [5, 6]. Unfortunately, outcomes in HFpEF are poor and could be compared to those of HFrEF, with 1-year mortality ranging between 10 and 30% [7]. A subanalysis of I-Preserve Trial (Irbesartan in Heart Failure With Preserved Ejection Fraction) showed (Table 1) that, in HFpEF, patients with diabetes have more signs of congestion, worse quality of life, higher levels of heart failure biomarkers (N-terminal pro-B-type natriuretic peptide: NT-proBNP), and a poorer prognosis [8]. In addition, comparing in-patient costs of heart failure admissions, patients with diabetes have the highest cost, and cost per day alive appears to be the highest for HFpEF patients with diabetes [9]. As already mentioned, the data for heart failure pharmacotherapy in HFpEF are so far very limited, and no trial has achieved convincing morbidity/mortality endpoints to date. This probably reflects the disease complexity, as there are multiple pathophysiologic mechanisms in HFpEF, such as impaired diastolic function and impaired systolic reserve, impaired longitudinal ventricular systolic and atrial function, impaired autonomic heart function, and peripheral mechanisms such as endothelial and skeletal muscle dysfunction [6, 10–13]. Now, the question is: “How should we treat patients with DHFpEF?”

## 3. “Established Heart Failure Drugs” in the Treatment of DHFpEF

### 3.1. Angiotensin-Converting Enzyme Inhibitors, Angiotensin Receptor Blockers, and Angiotensin Receptor/Neprilysin Inhibitors

Angiotensin-converting enzyme inhibitors (ACEi) reduce morbidity and mortality in patients with HFrEF and represent currently the standard of HFrEF pharmacotherapy [3, 4]. The evidence for the use of ACEi in HFrEF is coming from multiple randomized controlled trials [14–16]. Angiotensin receptor blockers (ARB), drugs directly blocking angiotensin receptor 1, have similar hemodynamic effect to ACEi, with a lower risk of cough and angioedema (side effects frequently limiting the tolerability of ACEi therapy). ARB have been shown to reduce morbidity and mortality in patients with HFrEF, especially in those not tolerating ACEi [17–19]. Considering these data, one could assume that ACEi or ARB should be preferred for HFpEF. This assumption might be supported with beneficial effects of these agents, such as reduction of afterload, reduction of myocardial fibrosis and myocardial remodeling [20, 21], anti-inflammatory effect, and improvement of endothelial function [22]. Nevertheless, looking more closely to this issue, the current evidence for the use of ACEi or ARB in the treatment of HFpEF is limited to that coming from CHARM (Candesartan in Heart failure: Assessment of Reduction in Mortality and morbidity)-Preserved Trial [23]. This trial (Table 1) enrolled 3023 patients with symptomatic heart failure and left ventricular ejection fraction >40% who were randomly assigned to candesartan (*n* = 1514, target dose 32 mg once daily) or matching placebo (*n* = 1509). The primary outcome of this trial was cardiovascular death or need for in-hospital admission for heart failure; patients were followed for a median of 36.6 months. In this trial, cardiovascular death did not differ between candesartan-treated patients and controls, but candesartan therapy reduced the need for in-hospital admissions (230 versus 279, *p* = 0.017). Although 28.7% of patients in candesartan group and 28.0% of patients in placebo group had a history of diabetes, the subanalysis of trial results in diabetic patients was not reported. On the other side, in the I-Preserve Trial [24], irbesartan therapy failed to improve the outcomes of HFpEF. In this trial enrolling 4128 patients with HFpEF, who were followed for a median of 49.5 months, the primary outcome occurred in 742 patients in the irbesartan group and in 763 patients in the placebo group, which was not statistically significant. Overall rates of death were 52.6 and 52.3 per 1000 patient-years, respectively (*p* = 0.98); rates of hospitalization for cardiovascular causes were 70.6 and 74.3 per 1000 patient-years, respectively (*p* = 0.44). The study groups did not differ significantly in diabetes status (27% of patients in irbesartan group and 28% of patients in placebo group), and similarly with previous trial, a subanalysis of study results in patients with diabetes was not specifically reported. Additionally, the Perindopril in Elderly People with Chronic Heart Failure Trial (PEP-CHF) was a randomized, placebo controlled, double-blind trial, which aimed to compare perindopril (dosed 4 mg/day) versus placebo in patients aged ≥70 years who were diagnosed to have heart failure (on diuretics) and an echocardiographic finding suggesting diastolic dysfunction and excluding substantial LV systolic dysfunction (LV EF > 40%) or valve heart disease [25]. The primary endpoint of this trial was a composite of all-cause mortality and unplanned heart failure-related hospitalization with a minimum follow-up of 1 year. In this trial, by the 1 year of follow-up period, reductions in the primary outcome (hazard ratio: HR 0.692, 95% confidence interval: CI 0.474-1.010; *p* = 0.055) and hospitalization for heart failure (HR 0.628, 95% CI 0.408-0.966; *p* = 0.033) were observed with perindopril. Furthermore, a functional class (*p* < 0.030) and 6-minute corridor walk distance (*p* = 0.011) had improved in those assigned to perindopril. Nevertheless, the enrollment and event rates in this study were lower than anticipated, which significantly reduced the power of the study to show a difference in the primary endpoint (to 35%). A significant amount of patients withdrew from study drugs (28% of patients taking perindopril and 26% of patients taking placebo) after 1 year and started taking open-label ACEi. The authors concluded that although improved symptoms and exercise capacity and fewer hospitalizations for heart failure in the first year were observed on perindopril, the study had insufficient power for its primary endpoint. The subanalysis of patients with diabetes was not reported (Table 1). Summarizing these data, currently, there is no clear evidence supporting the use of ACEi or ARB for the treatment of DHFpEF (as there are no data on global benefit of these agents in HFpEF from CHARM-Preserved and I-Preserve trials); however, looking on possible benefits, the therapy (preferring candesartan) could be probably considered in those patients with hypertension, left ventricular hypertrophy, prior myocardial infarction, in case of microalbuminuria/proteinuria, and diabetic kidney disease [4, 26]. Angiotensin receptor/neprilysin inhibitors, an ARB combined with a neprilysin inhibitor, commercially available as sacubitril/valsartan molecule, adds additional effect on heart failure by inhibition of enzyme which degrades natriuretic peptide, adrenomedullin, and other vasoactive peptides, leading to vasodilation and decreased retention of sodium [27]. The benefit of ARNI (compared to ACEi enalapril) in patients with HFrEF was demonstrated in PARADIGM-HF (Prospective Comparison of Angiotensin Receptor–Neprilysin Inhibitor with Angiotensin-Converting–Enzyme Inhibitor to Determine Impact on Global Mortality and Morbidity in Heart Failure) trial [28] which showed significant (by 20%) reduction of the composite endpoint of cardiovascular death or heart failure hospitalization with ARNI. ARNI therapy seemed promising in patients with HFpEF, considering the data from phase 2 clinical trial [29] which showed in patients with HFpEF a greater reduction of heart failure biomarkers with ARNI than with valsartan alone, and promising data from studies on animal models [30, 31] or data regarding beneficial effect of ARNI on left ventricular diastolic function coming from small postmarketing study [32]. However, these promises were not verified in PARAGON-HF (The Prospective Comparison of ARNI with ARB Global Outcomes in HF with Preserved Ejection Fraction) trial [33], a randomized trial comparing the effect of ARNI (sacubitril/valsartan) and valsartan alone in patients (with or without diabetes) with HFpEF (defined as left ventricular ejection fraction ≥ 45%) on a composite end-point of total hospitalizations for heart failure and death from cardiovascular causes. The study randomized totally 4822 patients with symptomatic HFpEF (left ventricular ejection fraction ≥ 45%), from whom 42.2% had diabetes. In this trial (Table 1), ARNI administration did not result in a significantly lower rate of total hospitalizations for heart failure and was not connected with significantly lower cardiovascular mortality (a trend towards benefit in reduction of primary events was observed, but the differences did not reach statistical significance: 894 primary events in the ARNI group versus 1009 primary events in the valsartan group; HR 0.87; 95% CI: 0.75-1.01; *p* = 0.06). Unfortunately, the subanalysis of trial outcomes in diabetic patients was not reported, and there is no other study reporting the efficacy and safety of ARNI for the treatment of DHFpEF. Undoubtedly, such a subanalysis/study could be off clinical interest, as ARNI could have in individuals with diabetes positive effect on glycaemic control [34] and renal function [35], suggesting a possible pleiotropic benefit for diabetic patients with heart failure. Nevertheless, the administration of ARNI still remains reserved for patients with HFrEF.

### 3.2. Mineralocorticoid Receptor Antagonists and Diuretics

Mineralocorticoid (aldosterone) receptor antagonists (MRA)—spironolactone and eplerenone—are recommended in all symptomatic patients with HFrEF to reduce mortality and heart failure-related hospitalizations [3]. The role of these agents in HFpEF is being intensively studied, with conflicting results. First, Deswal et al. reported in their small randomized study enrolling 44 patients with HFpEF that eplerenone therapy lead to a significant reduction in markers of collagen turnover and to an improvement in left ventricular diastolic function. However, in this trial, eplerenone did not improve exercise capacity during 6-minute walking test [36]. The TOPCAT (Treatment of Preserved Cardiac Function Heart Failure with an Aldosterone Antagonist) trial [37] examined the effect of spironolactone (dosed 15 to 45 mg daily) versus placebo on a composite endpoint of death from cardiovascular causes, aborted cardiac arrest, and hospitalization for heart failure. The study (Table 1) randomized totally 3445 patients with symptomatic heart failure and left ventricular ejection fraction ≥ 45%; from these patients, 32.5% had diabetes. Although spironolactone therapy in this study failed to improve primary composite endpoint (18.6% in spironolactone group versus 20.4% in placebo group, *p* = 0.14), patients treated with spironolactone had significantly lower incidence of hospitalizations for heart failure (12.0% versus 14.2%; *p* = 0.04). Based on these data, the American College of Cardiology formed its current recommendation on the use of MRA in HFpEF which states that MRA therapy might be considered in appropriately selected patients with HFpEF (left ventricular ejection fraction ≥ 45%, elevated markers of heart failure, heart failure-related hospitalization within 1 year, estimated glomerular filtration rate > 30 mL/min., and blood potassium level < 5 mmol/L) to decrease heart failure-related hospital admissions [4]. Looking on DHFpEF, a subanalysis of TOPCAT trial [38] showed that diabetic patients enrolled in this trial had higher levels of cardiac, profibrotic, and proinflammatory biomarkers. The administration of spironolactone in patients with diabetes appeared to improve the markers of extracellular matrix remodeling in an antifibrotic fashion. Additionally, Brandt-Jacobsen et al. [39] reported that the addition of high-dose eplerenone in patients with T2D and high risk for cardiovascular diseases was associated with a clear reduction in left ventricular mass and a clear reduction of NT-proBNP levels. Based on these data, MRA therapy could be probably recommended in selected patients with DHFpEF; however, due to diabetic-related kidney impairment, it might be more difficult to select appropriate DHFpEF patients for this therapy. Loop diuretics (furosemide, bumetanide, and torasemide) are recommended to reduce signs and symptoms of congestion in patients with HFrEF and HFpEF [3], but their effects on mortality and morbidity have not been studied in randomized trials. This recommendation should be probably applied also to DHFpEF patients, as there is no other pharmacologic approach to relieve signs and symptoms of congestion in HFpEF. However, the risk of side effects of the therapy, mainly the risk of orthostatic hypotension, worsening of renal function, and the risk of loss of minerals, could be higher in patients with diabetes [40].

### 3.3. Beta Blockers, Ivabradine, and Digoxin

A previous subanalysis of already mentioned I-PRESERVE trial showed that in this trial, heart rate (in sinus rhythm) was an independent predictor of adverse clinical outcomes. Each standard deviation (12.4 beats per minute) increase in heart rate was associated with an increase in risk of 13% for cardiovascular death or heart failure hospitalization (*p* = 0.002). Considering the data, “optimal” heart rate achieved with beta blockers or If-inhibition with ivabradine might be a “therapeutic target” in HFpEF. However, data regarding the use of beta blockers for HFpEF are inconsistent. The SENIORS (Study of Effects of Nebivolol Intervention on Outcomes and Rehospitalization in Seniors With Heart Failure) trial showed an overall benefit of beta blockage with nebivolol compared to placebo in 2128 heart failure patients > 70 years of age. The primary outcome was similar in the impaired and preserved left ventricular ejection fraction groups. However, the preserved left ventricular ejection fraction (HFpEF) was in this trial defined as left ventricular ejection fraction > 35% (and impaired left ventricular ejection fraction as ejection fraction ≤ 35%) [41]. On the other side, in the Japanese Diastolic Heart Failure Study (J-DHF), carvedilol therapy did not improve the prognosis of HFpEF patients (Table 1) [42]. In contrast, data from the Croatian heart failure registry showed a higher overall survival rate, improvement in ejection fraction, and NYHA class in HFpEF patients on long-term (at least 4 years) carvedilol therapy [43]. None of these studies was dedicated on patients with DHFpEF, and there is no other study examining the effect of beta blockage on clinical outcomes in those with DHFpEF. Therefore, routine beta blockage in patients with DHpEF is currently not recommended. However, beta blockers might be considered in patients with DHFpEF and arterial hypertension or known coronary artery disease [4, 26]. If-inhibition with ivabradine improved vascular stiffness, left ventricular contractility, and diastolic function in a mouse model of HFpEF [44]. Moreover, ivabradine therapy improved diastolic left ventricular function in an observational study performed by Cacciapuoti et al. [45] and significantly reduced the need of hospital admissions for worsening heart failure in a previous randomized placebo-controlled study enrolling patients with HFrEF [46]. The effect of ivabradine therapy on clinical outcomes in patients with HFpEF was specifically examined in the EDIFY (prEserveD left ventricular ejectIon fraction chronic heart Failure with ivabradine study) trial [47]. This randomized, placebo-controlled trial (Table 1) included 179 patients with symptomatic (NYHA classes II and III) HFpEF (left ventricular ejection fraction 45%), who were in sinus rhythm (with heart rate of ≥70 beats per minute) and had an elevation of heart failure biomarkers (NT-proBNP of ≥220 pg/mL or BNP ≥ 80 pg/mL). Patients were randomized to ivabradine (uptitrated to 7.5 mg b.i.d.) or placebo and followed for 8 months for the incidence of primary end-point defined as improvement in left ventricular diastolic function (assessed by echocardiography), distance on the 6-minute walking test, and change of plasma NT-proBNP levels. In this trial, ivabradine did not improve any of the three clinical outcomes (although there was a significant decrease in heart rate by ivabradine therapy). 43.2% of patients randomized to ivabradine and 44.1% of patients randomized to placebo had diabetes; the subanalysis of outcomes among patients with diabetes was not reported. Additionally, there is no other study examining the efficacy/safety profile of ivabradine in patients with DHFpEF. Summarizing, although several small studies pointed on a possible positive effect of heart rate reduction with ivabradine in patients with HFpEF, based on the results of EDIFY trial, ivabradine is not indicated for the treatment of HFpEF/DHFpEF. Thus, ivabradine should not be administrated in diabetic patients with HFpEF, unless there is other indication for ivabradine therapy (such as symptomatic angina despite beta blocker therapy). Digoxin might be considered as a treatment option in patients with HFrEF in sinus rhythm who remain symptomatic despite ACEi (or ARB), a beta blocker, and MRA therapy to reduce the risk of hospitalizations, or in patients with HFrEF and atrial fibrillation to slow a rapid ventricular rate [3]. Currently, there is no randomized study examining the efficacy/safety profile of digoxin in the treatment of HFpEF/DHFpEF. Furthermore, in a previously published analysis of a heart failure registry, the initialization of digoxin therapy in patients with HFpEF requiring heart failure-related hospitalization prior their discharge was not associated with lower rates of rehospitalizations or all-cause mortality [48]. Moreover, data from another observational multicentre study suggested that digoxin therapy in patients with HFpEF might be associated with increased mortality and/or heart-failure-related readmission, especially in patients with lower heart rate [49]. Therefore, digoxin is not indicated in patients with HFpEF, and this could be probably applied also to patients with DHFpEF.

## 4. Novel Antidiabetic Drugs: Promising Agents for the Treatment of DHFpEF?

### 4.1. Sodium-Glucose Cotransporter 2 Inhibitors

The data regarding significant benefit of sodium-glucose cotransporter 2 (SGLT-2) inhibition on heart failure-related mortality and heart failure-related hospitalizations came firstly from studies which were not specifically dedicated on heart failure patients. The EMPA-REG OUTCOME (Empagliflozin Cardiovascular Outcome Event Trial in Type 2 Diabetes Mellitus Patients) trial [50] randomized a total of 7020 patients with T2D at high cardiovascular risk (left ventricular ejection fraction was not reported) to receive 10 mg or 25 mg of empagliflozin or placebo once daily. The primary composite outcome was death from cardiovascular causes, nonfatal myocardial infarction, or nonfatal stroke, as analyzed in the pooled empagliflozin group versus the placebo group. The primary outcome occurred in 10.5% of patients in the pooled empagliflozin group and in 12.1% of patients in the placebo group (*p* = 0.04 for superiority). In the empagliflozin group, there were significantly lower rates of death from cardiovascular causes (3.7%, versus 5.9%; 38% relative risk reduction), hospitalization for heart failure (2.7% versus 4.1%, 35% relative risk reduction), and death from any cause (5.7% versus 8.3%, 32% relative risk reduction). The study showed cardiovascular benefit of SGLT2 inhibition in T2D patients; there was no specific mention of HF or left ventricular ejection fraction status in this study. Similar data in patients with T2D and higher risk of cardiovascular diseases were subsequently published with dapagliflozin [51] and with canagliflozin [52]. The EMPEROR-Reduced (EMPagliflozin outcomE tRial in Patients With chrOnic heaRt Failure With Reduced Ejection Fraction) trial [53] tested the effect of empagliflozin (10 mg once daily) on a composite end-point of cardiovascular death or hospitalization for worsening heart failure in patients with symptomatic HFrEF (left ventricular ejection fraction ≤ 40%). This study randomized totally 3730 patients (with or without diabetes) who were followed for a median of 16 months. The primary outcome was less frequent in the empagliflozin group (19.4% versus 24.7%, *p* < 0.001). The effect of empagliflozin on the primary outcome was consistent in patients regardless of the presence or absence of diabetes. The total number of hospitalizations for heart failure was lower in the empagliflozin group than in the placebo group. In addition, empagliflozin-treated patients had lower risk of serious renal outcomes. The study confirmed significant cardiovascular benefit of SLGT2 inhibitor therapy in a cohort of patients with HFrEF, regardless of T2D status. Another robust evidence for the use of SGLT-2 inhibition in patients with HFrEF comes from DAPA-HF (Study to Evaluate the Effect of Dapagliflozin on the Incidence of Worsening Heart Failure or Cardiovascular Death in Patients With Chronic Heart Failure) trial [54]. This randomized, placebo-controlled trial enrolled 4744 patients (with or without diabetes) with symptomatic heart failure and reduced left ventricular ejection fraction (≤40%) who were randomized to receive either dapagliflozin (10 mg once daily) or placebo, in addition to standard of care therapy. Patients were followed for a median of 18.2 months for the incidence of primary outcome defined as a composite of worsening heart failure (hospitalization or an urgent visit resulting in intravenous therapy for heart failure) or cardiovascular death. During the follow-up period, the primary outcome occurred in 16.3% of patients in the dapagliflozin group and in 21.2% of patients in the placebo group (*p* < 0.001). A first worsening heart failure event occurred in 10.0% of patients in the dapagliflozin group and in 13.7% of patients in the placebo group; cardiovascular disease-related death occurred in 9.6% of patients in the dapagliflozin group and in 11.5% of patients in the placebo group, respectively. In addition, results in diabetic patients were similar to those in patients without diabetes. Although promising, the results of upper-mentioned studies cannot be directly applied to patients with DHFpEF. Therefore, it is not surprising that EMPEROR Preserved (EMPagliflozin outcomE tRial in Patients With chrOnic heaRt Failure With Preserved Ejection Fraction) trial [55] and DELIVER (Dapagliflozin Evaluation to Improve the LIVEs of Patients With PReserved Ejection Fraction Heart Failure) trial [56], studies directly dedicated on patients with (D)HFpEF, have been designed. The DELIVER (Dapagliflozin Evaluation to Improve the LIVEs of Patients With PReserved Ejection Fraction Heart Failure) trial [57] is designed as an international, multicentre, randomized, placebo-controlled study which aims to test the effect of SGLT-2 inhibition with dapagliflozin (10 mg daily) versus placebo, in addition to standard of care, in patients with HFpEF (left ventricular ejection fraction ≥ 40%). Study (Table 1) plans to enroll patients with or without diabetes (6263 patients), with preserved ejection fraction, with signs and symptoms of heart failure, elevation in natriuretic peptides, and evidence of structural heart disease. The primary endpoint will be the time-to-first cardiovascular death or worsening heart failure event (heart failure hospitalization or urgent heart failure visit). Finally, the results of the EMPEROR-Preserved (EMPagliflozin outcomE tRial in Patients With chrOnic heaRt Failure With Preserved Ejection Fraction) trial [55] have been recently reported [57]. The study was designed to test the SGLT-2 inhibition with empagliflozin in patients with HFpEF. The study (Table 1) enrolled 5988 patients with class II–IV heart failure and left ventricular ejection fraction > 40%, with and without T2D, who were randomized to receive empagliflozin (10 mg daily) or placebo, in addition to standard therapy. In this study, 2997 patients received empaliflozin and 2991 patients received placebo. The primary end-point of this study was a composite of cardiovascular death or hospitalization for heart failure. In a median of 26.2 months of clinical follow-up, the primary outcome occurred in 13.8% in the empagliflozin group and in 17.1% in the placebo group (hazard ratio, 0.79; 95% confidence interval [CI]: 0.69-0.90; *p* < 0.001). This effect was mainly related to a lower risk of hospitalization for heart failure. The efficacy of empagliflozin was consistent in patients with or without T2D. The total number of hospitalizations for heart failure was lower in the empagliflozin group than in the placebo group (407 with empagliflozin versus 541 with placebo; HR 0.73; 95% CI: 0.61-0.88; *p* < 0.001). Looking on the side effects of the therapy, unsurprisingly, uncomplicated genital and urinary infections and hypotension were reported more frequently with empagliflozin. The results of the EMPEROR-Preserved trial confirmed the efficacy of empagliflozin in patients with HFpEF (with and without T2D) and will probably establish SGLT-2 inhibition as a first evidence-based and clinical practice guideline-recommended pharmacologic therapy for HFpEF and also for DHFpEF.

To summarize, empagliflozin is the first agent with evidence-based efficacy data for the treatment of HFpEF (irrespectively on diabetes status). Based on a robust evidence in T2D patients with higher cardiovascular risk, in patients with HFrEF, as well as in those with HFpEF, SGLT-2 inhibitors should be administrated in patients with DHFpEF (unless contraindicated) to treat diabetes (to safely improve glycaemic control), to reduce the risk of heart failure-related hospitalizations and cardiovascular mortality and to improve renal outcomes.

### 4.2. Glucagon-Like Peptide 1 Receptor Antagonists

Glucagon-like peptide 1 receptor antagonists (GLP-1 RA), such as liraglutide, semaglutide, or dulaglutide, are novel antidiabetic agents with the evidence for reduction of cardiovascular events [26]. In previous randomized controlled trials in patients with T2D, liraglutide reduced the rate of the first occurrence of death from cardiovascular causes, nonfatal myocardial infarction, or nonfatal stroke [58], semaglutide significantly reduced the rate of cardiovascular death, nonfatal myocardial infarction, or nonfatal stroke [59], and dulaglutide reduced the primary outcome defined as the first occurrence of the composite endpoint of nonfatal myocardial infarction, nonfatal stroke, or death from cardiovascular causes [60]. Nevertheless, the cardiovascular benefit of GLP-1 RA was mostly due to reduction of adverse vascular events and reduced cardiovascular mortality and not due to reduced incidence of heart failure or reduced rate of heart failure-related hospitalizations. Additionally, none of these studies were dedicated on a population of patients with diabetic heart failure. Withaar et al. previously demonstrated in a study using HFpEF animal model that treatment with liraglutide improved the cardiometabolic dysregulation and cardiac function, reduced cardiac hypertrophy, reduced myocardial fibrosis, and improved atrial weight, natriuretic peptide levels, and lung congestion [61]. In another animal model, liraglutide improved pressure-overload induced cardiac hypertrophy and cardiac apoptosis [62], and liraglutide administration had favorable effects on BNP and left ventricular diastolic function, but not on left ventricular ejection fraction, in a small-sample clinical study in patients with T2D and preserved left ventricular ejection fraction performed by Yagi et al. [63]. Nonetheless, there is no clinical study examining the effect of GLP-1 RA therapy on clinical outcomes in patients with DHFpEF (reported or on-going). Thus, GLP-1 RA cannot be considered as a treatment option for DHFpEF. However, it is probably rational to administer GLP-1 RA in patients with T2D and known cardiovascular disease (including heart failure), especially in those with known coronary artery disease or other known atherosclerotic vascular diseases, to treat diabetes (to safely improve glycaemic control), and to prevent future vascular events.

## 5. Conclusion

How to treat DHFpEF? Right now, there is no conclusive answer to this question. Obviously, there is a need to treat the cause of the HFpEF (if treatable) and manage comorbidities as is currently recommended in HF guidelines (T2D is a common comorbidity in HFpEF) [3, 64]. In addition, considering the discussed data, it is probably reasonable to administer loop diuretics in those patients with signs or symptoms of congestion; ARB in patients with left ventricular hypertrophy, hypertension, or concomitant diabetic kidney disease and SGLT-2 inhibitors in all reliable patients with DHFpEF to treat diabetes, reduce the risk of heart failure-related hospitalizations and cardiovascular mortality, and improve renal outcomes. Additionally, selected patients with DHFpEF could benefit from beta blocker and MRA therapy. Moreover, it is probably rational to administer GLP-1 RA in patients with T2D and known cardiovascular (especially atherosclerotic) disease to treat diabetes and prevent future vascular adverse events. Nevertheless, the issue of optimal pharmacotherapy for DHFpEF is still open for future research.

## Figures and Tables

**Table 1:** Summary of studies on heart failure with preserved ejection fraction.

Stud**y**	Study size	Patient population	Dedication on diabetes	Studied drug and comparator	Main outcomes	Conclusion
CHARM–Preserved [23]	3023 patients	NYHA functional class II-IV CHF and LVEF > 40%	No (857 patients with diabetes; subanalysis was not reported)	Candesartan (1514 patients, target dose 32 mg once daily) or matching placebo (1509 patients)	22% patients in the candesartan vs. 24% in the placebo group experienced the primary outcome (*p* = 0.051); no reduction of CV death; fewer patients with candesartan group admitted to hospital for CHF	Moderate impact in preventing admissions for CHF
I–Preserve [24]	4128 patients	At least 60 years of age, NYHA class II-IV HF and LVEF ≥ 45%	No (1134 patients with diabetes; subanalysis was not reported)	300 mg of irbesartan (2067 patients) or placebo (2061 patients)	Primary event rates with irbesartan 100.4 vs. 105.4 per 1000 patient-years with placebo (*p* = 0.35); no reduction of CV death or CVD-related hospitalization	No impact on outcomes
PEP–CHF [25]	850 patients (207 patients finished follow-up)	At least 70 years of age, HF with diastolic dysfunction, LVEF > 40%	No (175 patients with diabetes; subanalysis was not reported)	4 mg of perindopril (424 patients; 100 evaluated) or placebo (426 patients, 107 evaluated)	Reduction in the primary outcome, HF-related hospitalizations, NYHA class and 6-MWT distance	Significant impact on outcomes; however, insufficient power of study for primary endpoint
PARAGON–HF [33]	4822 patients	NYHA class II-IV HF, LVEF ≥ 45%, elevated level of natriuretic peptides, evidence of structural heart disease	No (2062 patients with diabetes; subanalysis was not reported)	Sacubitril-valsartan (2407 patients, target dose, 97 mg of sacubitril with 103 mg of valsartan twice daily) or valsartan (2389 patients, target dose, 160 mg twice daily)	894 primary events in 526 patients in the sacubitril-valsartan group and 1009 primary events in 557 patients in the valsartan group (*p* = 0.06); no reduction of CV death or HF-related hospitalization	No impact on outcomes
TOPCAT [37]	3445 patients	Symptomatic HF and LVEF ≥ 45%	No (1118 patients with diabetes; subanalysis was not reported)	Spironolactone (1722 patients, 15 to 45 mg daily) or placebo (1723 patients)	Primary outcome occurred in 18.6% of patients with spironolactone vs. 20.4% of patients with placebo (*p* = 0.14); no reduction of CV death or hospitalization (for any reason)	No impact on outcomes
J–DHF [42]	245 patients	At least 20 years of age, HF and LVEF >40%	No (75 patients with diabetes; subanalysis was reported)	Carvedilol (120 patients, uptitrated from 1.25 mg twice daily to the target dose of 10 mg twice daily) or no carvedilol (125 patients)	Primary endpoint occurred in 29 patients in the carvedilol group vs. in 34 patients in the control group (*p* = 0.68); no reduction of CV death, HF-related or CVD-related hospitalization (no difference according to diabetes status)	No impact on outcomes (similar in patients with and without diabetes)
EDIFY [47]	179 patients	NYHA class II-III, in sinus rhythm with HR of ≥70 b.p.m., NT-proBNP level of ≥220 pg/mL (or BNP ≥ 80 pg/mL) and LVEF ≥ 45%	No (78 patients with diabetes; subanalysis was not reported)	Ivabradine (95 patients, titrated to 7.5 mg b.i.d.) or placebo (84 patients)	No evidence of improvement was found in any of the three coprimary endpoints (Doppler *E*/*e*′ ratio, distance on the 6MWT, and decrease of plasma NT-proBNP levels)	No impact on outcomes
EMPEROR Preserve [55, 57]	5988 patients	NYHA class II-IV HF and LVEF > 40%	No (2934 patients with diabetes; subanalysis was reported)	Empagliflozin (2997 patients, 10 mg once daily) or placebo (2991 patients)	Primary outcome event occurred in 13.8% of patients with empagliflozin vs. in 17.1% of patients with placebo (*p* < 0.001); the effect mainly related to a lower risk of HF-related hospitalization (no difference according to diabetes status)	Significant reduction of the combined risk of CV death or hospitalization for HF(similar in patients with and without diabetes)
DELIVER [56]	6263 patients	At least 40 years of age, CHF and LVEF > 40%	No (patients with or without diabetes enrolled; subanalysis will be probably reported)	Dapagliflozin (10 mg once daily) or placebo	Ongoing study	Ongoing study

6MWT: 6 min walking test; BNP: B-type natriuretic peptide; (C)HF: (chronic) heart failure; CV(D): cardiovascular (disease); HR: heart rate; NYHA: New York Heart Association.

## Data Availability

All the source data are available at the corresponding author upon a reasonable request.
